# Multifunctional Nanocomposite Cellulose Fibers Doped in Situ with Silver Nanoparticles

**DOI:** 10.3390/polym11030562

**Published:** 2019-03-25

**Authors:** Olga Rac-Rumijowska, Irena Maliszewska, Marta Fiedot-Toboła, Iwona Karbownik, Helena Teterycz

**Affiliations:** 1 Faculty of Microsystem Electronics and Photonics, Wrocław University of Technology, Janiszewskiego 11/17, 50-372 Wrocław, Poland; helena.teterycz@pwr.edu.pl; 2 Faculty of Chemistry, Wrocław University of Technology, Norwida 4/6, 50-373 Wrocław, Poland; irena.helena.maliszewska@pwr.edu.pl; 3 Polish Centre for Technology Development PORT, Stabłowicka 147, 54-066 Wroclaw, Poland; marta.fiedot-tobola@eitplus.pl; 4 Faculty of Electrical, Electronic, Computer and Control Engineering, Technical University of Łódź, Żeromskiego 116, 90-924 Łódź, Poland; ivakabari@gmail.com

**Keywords:** silver nanoparticles, cellulose fibers, nanocomposite fibers, antibacterial properties, flame resistivity

## Abstract

This paper presents a method for the preparation of nanocomposite cellulose fibers doped with silver nanoparticles (AgNPs), as well as the effect of silver nanoparticles on the structure and properties of fibers. The fibers were obtained by an environmentally friendly method using N-Methylmorpholine N-oxide (NMMO) as a solvent, in a non-polluting closed system. Doping with silver nanoparticles was carried out as a direct (in situ) reduction of Ag^+^ ions in the presence of a stabilizing agent during the preparation of the spinning solution. SEM images of the surface and cross section of the fibers showed that the distribution of nanoparticles in the fibers’ volume was uniform. The fibers exhibited very good antibacterial properties in relation to *Staphylococcus aureus*, *Escherichia coli*, *Acinetobacter baumannii*, and *Candida albicans*. Flammability analysis showed that the fibers were subjected to a one-stage combustion process and that the silver nanoparticles reduced the heat release rate (HRR) of the fibers by 36%. TG studies showed that the modification of cellulose fibers with silver nanoparticles promoted the formation of mill scale in the combustion of fibers, which was directly related to the reduction of flammability. Tests of the electrical properties showed that the linear resistance of cellulose fibers containing 3 wt % silver was 10^8^ Ω/cm.

## 1. Introduction

There is currently a great demand for textile materials with new properties. It is increasingly required that fibers or fabrics not only have the traditional protective or decorative functions but also have a number of new properties, e.g., antibacterial [1,2], photocatalytic [3], or flammability [4] properties. The most common method of changing the properties of a fiber is the surface modification of finished products with compounds such as ZnO [5] or a mixture of it with TiO_2_ [6], in order to obtain multifunctional textile materials with greater surface softness, improved protection against UV radiation, and antibacterial properties. One commonly used compound for fabric modification is silver nanoparticles. The high interest in this compound is due to the effective bactericide and fungicide activities of silver, which allow to use a small amount of it, compared to zinc oxide, to obtain a material with very good antimicrobial properties [7]. Silver is most often added to give materials biocidal properties [8], biopolymer stabilization [9], an increase in their thermal stability [10] and electrical conductivity [11], as well as new optical properties [12]. The color of polymer-silver nanocomposites varies from yellow to orange or brown, depending on the concentration and size of the applied nanoparticles. This is due to the optical properties of silver nanoparticles in which localized surface plasmon resonance occurs. Most accidents involving the modification of textiles with silver nanoparticles relies on the direct reduction of silver ions on the surface of materials such as cotton [13], jute fabrics [14], or viscose fibers [15], for which silver nanoparticles are used to confer them antibacterial properties or to increase their electrical conductivity [16].

To reduce the possibility of losing the surface layers, the optimal method of modifying textile materials seems to be a composite method. Because of the thinness of fibers, the use of nanomaterials as fillers is the most effective method. The biggest challenge in the production of nanocomposites, especially in the form of fibers, is the achievement of a good degree of dispersion of the filler. However, because of the high surface energy of nanoparticles, it is almost impossible to obtain a very high dispersion using dry nanopowders [17], which is particularly evident in the case of conductive fibers. In these composites, exceeding the threshold of percolation, which is the point at which electrical conductivity increases, is directly related to the weakening of mechanical strength. This is due to the fact that exceeding this threshold is associated with the addition of large quantities of filler from a few to even several dozen vol % units [18]. For this reason, it is necessary to develop new methods to obtain nanocomposites. Fakirov [19] presented the concept of converting instead of adding, presenting the possibility of producing a polymer nanocomposite with excellent dispersion of the filler in the matrix as a result of the production process. The method presented in this paper is based on this concept. This method consists of obtaining nanocomposite fibers as a result of direct synthesis of silver nanoparticles in a spinning solution. In our previous paper [20], we presented a detailed procedure for obtaining silver nanoparticles in an NMMO-based spinning solution, as well as a preliminary fiber characterization. In this work, the impact of nanoparticles on the structure of fibers, as well as the multifunctional properties of the obtained nanocomposite fibers, is presented.

## 2. Materials and Methods 

### 2.1. Materials

Cellulose pulp containing 98 wt % α-cellulose with an average degree of polymerization (DP) of about 1250 was used for the preparation of cellulose fibers. A 50% water solution of N-Methylmorpholine N-oxide (NMMO) was purchased from Hustman Co (The Woodlands, TX, USA). The anti-oxidant propyl ester of gallic acid (Tenox PG) was purchased from Aldrich (Sigma-Aldrich Co., St. Louis, MO, USA). The precursor of silver ions was silver nitrate (AgNO_3_) from POCH (POCH, Gliwice, Poland), and the stabilizing agent of nanoparticles was polyethyleneimine (PEI) MW 2 kDa from Aldrich (Sigma-Aldrich Co., St. Louis, MO, USA). All the above-mentioned chemicals were used as received without further purification.

### 2.2. Preparation of Sellulose Fibers

Nanocomposite cellulose fibers were obtained as a result of the synthesis of silver nanoparticles (AgNPs) in situ in the spinning dope, which was previously described by the authors [20]. The spinning dopes were made using a laboratory scale knitter (IKA, Warsaw Poland), and a small spinning device was used for the preparation of cellulose fibers. The cellulose fibers were obtained using the NMMO method (lyocell process). The spinning solution was obtained by dissolving cellulose in a 50% aqueous solution of NMMO in the presence of an antioxidant in the amount of 1% wt in relation to the amount of cellulose. In the case of nanocomposite fibers, silver nitrate (0.1–3% wt for cellulose content) and a 1M aqueous solution of PEI (molar ratio Ag/PEI = 1:8) were added to the crusher. The obtained mixture was stirred and heated until a homogeneous solution of cellulose was obtained. Excess water was removed under reduced pressure. The fibers were formed using the wet–wet method by pressing them through a nozzle of a heated solution. The fibers were solidified in an aqueous coagulation bath (cold) and then rinsed in a hot plastic bath and dried.

### 2.3. Structure and Morphology Analysis

The cross section of the fibers was observed and obtained using a high-resolution SEM Xe-PFIB FEI Helios (Brucker, Billerica, MA, USA). A single fiber was covered with a platinum layer, and then a cross section was made using a focus ion beam (FIB). The crystallographic structure of the obtained fibers was studied using a Philips Materials Research Diffractometer (Philips, Amsterdam, The Netherlands) with CuKa radiation. The θ/2θ scan, typical of powder materials, was used. The degree of crystallinity was determined on the basis of the optimization carried out in the WAXSFIT program. The chemical structure of the samples was examined by Fourier transform infrared spectroscopy (FTIR) using a Thermo Scientific Nicolet 380 spectrometer (Thermo Fisher Scientific, Waltham, MA, USA). Spectra were recorded in the range of 60–4000 cm^−1^ with a resolution of 4 cm^−1^.

### 2.4. Thermal Properties

Flammability tests were carried out using “Method A” according to the standard test method for determining flammability characteristics of plastics and other solid materials using microscale combustion calorimetry (ASTM:D 7309-07a) [21]. The temperature of the pyrolyzer was 75–750 ℃, the temperature increase was 1 ℃/s, the pyrolizer atmosphere was N_2_, the furnace temperature was 900 ℃, the furnace atmosphere N_2_/O_2_ was 80/20 cm^3^, and the total gas flow was 100 cm^3^/min. The sample weights were about 1 mg. Each fiber was measured three times, and the results were averaged. The tests were carried out on a FAA Micro Calorimeter device from FTT (Fire Testing Technology, Grinstead, UK) company.

Thermogravimetry studies (TG) were performed in the temperature range of 25–600 °C and at a heating rate of 5, 10, 15, 20 °C/min under nitrogen (30 mL/min) for the TGA2 Mettler Toledo Thermobalance (Columbus, OH, USA). Activation energy (Ea) at the decomposition temperature was determined using linear isoconversion methods (Kissinger–Akahira–Sunose (KAS) and Flynn–Wall–Ozawa (FWO)). In addition, changes in the Ea values in the function of material conversion were determined using the model-free kinetics method developed by Vyazovkin.

### 2.5. Determination of Antimicrobial Activity

The antibacterial properties of the modified textiles were determined using the serial dilution method [22] against four different pathogens: *Staphylococcus aureus* (PCM 458), *Escherichia coli* (PCM 2209), *Acinetobacter baumannii* (ATCC 19606) and *Candida albicans* (PCM 2566). An amount of 1 mL of an overnight culture of the tested microorganisms (grown aerobically at 37 °C with shaking in Mueller broth) was centrifuged at 6000× *g* for 3 min, and then the supernatant was discarded. The pellet was resuspended in 1 mL of saline to give an inoculum of approximately 1.3 × 10^6^ colony-forming units (CFU/mL) for *S. aureus* and *C. albicans*, and 1.3 × 10^7^ CFU/mL for *E. coli* and *A. baumannii*. Samples of the modified fibers (1 × 10^−3^ g, 2 × 10^−3^ g, 3 × 10^−3^ g, 4 × 10^−3^ g, and 5 × 10^−3^ g) were placed in tubes containing 3 mL of saline. Then, 300 (or 30) μL of pre-prepared bacterial suspension was added to the tubes, and they were then incubated for 5 h at 37 °C. Serial dilutions were then prepared, and 100 µL aliquots of each dilution was seeded in duplicate onto Mueller Agar (Difco) and incubated for 24–48 h at 37 °C. After incubation, the number of CFU/mL was established. The tests were carried out for each microorganism separately, and all assays were performed in duplicate and repeated two times. The percentage reduction of microorganisms after 24 h of incubation was calculated using the Formula (1):

(1) $$\frac{B-A}{B}\times 100,$$

where: *A* is the number of microorganisms (CFU/mL) in the tested sample after 5 h of incubation, *B* is the number of microorganisms (CFU/mL) estimated in the starting suspension before the addition of the fibers (time 0).

Microbial suspensions treated with unmodified cellulose fibers (without silver nanoparticles) as well as suspensions without the addition of cellulose samples were the positive and negative controls.

### 2.6. Electrical Conductivity

Electrical conductance was investigated by the spectroscopy impedance (SI) method using a Solartron SI 1260 analyzer (Solartron, Bognor Regis, UK). The measurement was carried out in the frequency range from 1 Hz to 1 MHz. The samples were stimulated by a sinusoidal voltage signal with an amplitude of 3 V. Measurements were made on a 1 cm length between two cooper electrodes. The electrical properties of the fibers were described by specifying the linear resistance value (Rl), which is the average resistance of a fiber with a length of one centimeter, given in Ω/cm.

### 2.7. Fastness Test to Washing

The washing process of nanocomposite fibers was carried out by using the home laundry washing method [23]. The washing solution was prepared by dissolving 2 g of Na_2_CO_3_ and 5 g of commercial laundry detergent, and the cellulose to solution weight ratio was 1:50. The fibers were immersed in a washing solution at 60 °C and mixed for 15 min. After washing, the fibers were rinsed in clean water and dried at 70 °C for 15 min. Silver content tests in the solutions after washing were made using the Atomic Absorption Spectrometry (AAS) method on a Thermo Scientific iCAP 7400 duo (BAD-00000348/001) spectrometer (Thermo Fisher Scientific, Waltham, MA, USA). After the washing test, the determination of the biocidal effectiveness of the cellulose fibers modified with 3% of AgNP was carried out in accordance with the protocol described above (see: Determination of antimicrobial activity); 3 × 10^−3^ g, 4 × 10^−3^ g, and 5 × 10^−3^ g of these fibers were used in the experiments.

## 3. Results and Discussion

**Figure 1:**
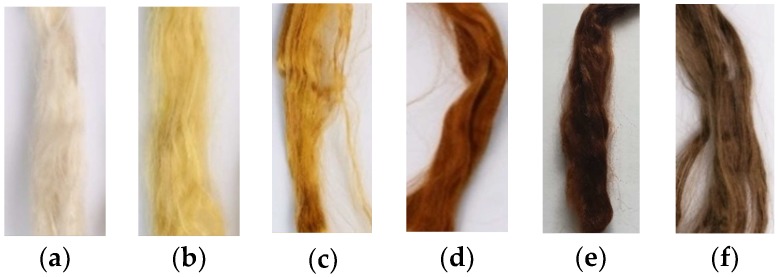
Photographs of cellulose fibers containing silver nanoparticles: (**a**) 0,01%, (**b**) 0,05%, (**c**) 0.1%, (**d**) 1%, (**e**) 3% synthesized in the presence of polyethyleneimine (PEI); (**f**) 0.1% synthesized without PEI.

The developed method of silver nanoparticles synthesis with a high concentration in the spinning solution allowed cellulose fibers with a high concentration of nanoparticles to be obtained. The cellulose fibers contained 0.01–3 wt % of AgNPs. Comparative fibers containing 0.1% of silver were also formed, but the process was carried out without a stabilizing agent (PEI). The presence of silver nanoparticles in the fibers resulted in a change in their color (Figure 1). Unmodified cellulose fibers are white, whereas the color of nanocomposite fibers depends on the concentration of silver and changed from slightly creamy (0.01 wt % Ag), to intensely yellow (0.1 wt % Ag) and then to brown (1 and 3 wt % Ag). The color of fibers with the same silver content, but not containing a stabilizing agent, was much darker, indicating that the particles had a much larger diameter (Figure 1f).

### 3.1. Microstructure of Fibers

**Figure 2:**
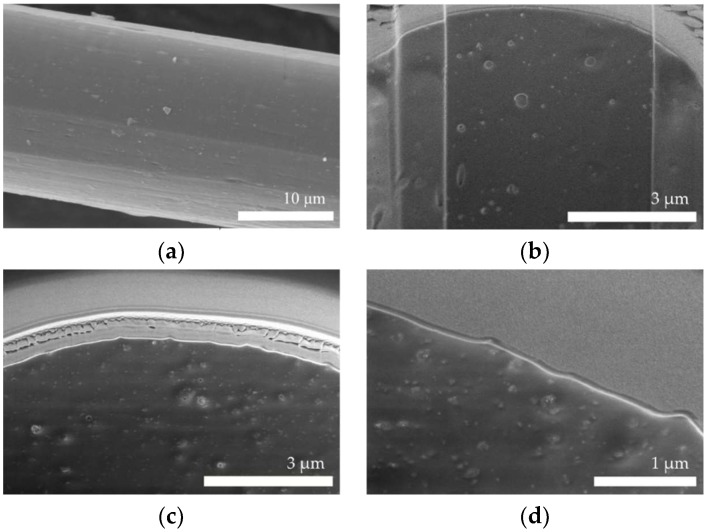
SEM images of cellulose fibers: (**a**) 3% of silver nanoparticles (AgNPs); cross section of: (**b**) undoped fibers (**c**,**d**) fibers containing 3% of AgNPs.

The surface of the nanocomposite fibers was smooth and did not contain agglomerates (Figure 2a). The cross section of the unmodified fiber was completely smooth (Figure 2b), and only the bubbles resulting from the preparation of the cross section of the sample were visible. On the cross section of the fiber containing 3% of silver nanoparticles, grainy precipitations of several nanometers were visible (Figure 2c,d).

### 3.2. Analysis of the Structural Properties (XRD)

**Figure 3:**
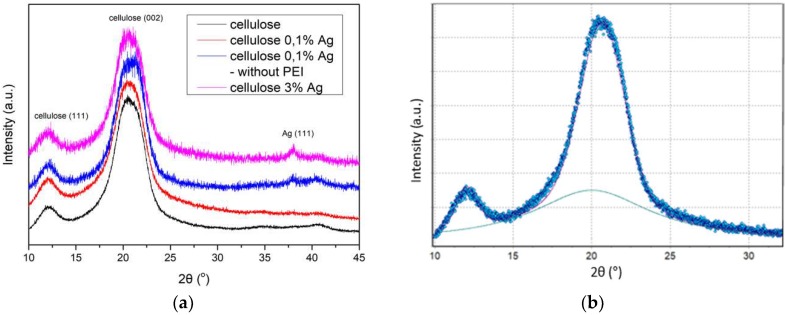
(**a**) XRD of cellulose fibers, (**b**) experimental curve with an amorphous area.

Analysis of the crystalline structure of the cellulose fibers and the study of the effect of silver nanoparticles on their structure were based on X-ray diffractograms (XRD) of both unmodified fibers and silver nanoparticles (Figure 3a). The effect of the nanoparticle stabilizer on the crystal structure was also determined. The degree of fiber crystallinity (*X_c_*) was calculated using the Hinrichsen method [24] (2):

(1) $$X_{c}=\left[\frac{I_{c}}{I_{c}+I_{a}}\right],$$

where *I_c_* is the integral under the peaks corresponding to the crystalline phase of the polymer, *I_a_* is the integral under the peaks corresponding to the amorphous phase of the polymer.

The crystalline and amorphous peaks were difficult to separate. For this reason, the diffraction curve was analyzed by creating a theoretical function that was best suited to the experimental curve. Curve fitting was performed using the Rosenbrock method [25]. The optimization was carried out with the WAXFIT program (Figure 3b) [26]. The average size of the fiber and nanoparticle crystallites was determined on the basis of the half-width of the 2θ peak, characteristic of specific structures, using the Debye–Scherrer Equation (3) [27]:

(3) $$L_{\left(hlk\right)}=\frac{K\lambda }{B\cos\theta_{max}}$$

where *L*_(*khl*)_ is the average size of crystallites, *K* is the Scherrer constant (0.89), *θ_max_* is the angle for the maximum peak [rad], *λ* is the length of the radiation beam [Å]; *β*—is half-width of the peak [rad].

The inter-chain separation length (*R*) was determined on the basis of the analysis of the most intense crystalline peak from the Equation (4) [28]:

(4) $$R=\frac{5\lambda }{8\sin\theta_{max}}$$

The diffractograms of the cellulose fibers showed characteristic peaks at angles of 2θ~12.4° (111) and 22.6° (002) (Figure 3a), and the diffractograms of the nanocomposite fibers showed the characteristic peaks at 38.1, corresponding to (111). This is characteristic of the face-centered cubic (fcc) structure of metallic silver nanoparticles.

The degree of crystallinity of pure cellulose fiber is 0.54, and that of cellulose fibers containing 0.1% of AgNPs was 0.71 (Table 1). The degree of crystallinity of fibers containing 0.1% of silver particles synthesized without a stabilizer remained unchanged. It follows that the presence of a small amount of nanoparticles that were well dispersed in the volume of cellulose fiber greatly facilitated the crystallization process. Silver particles, formed without the addition of a stabilizing agent, formed agglomerates and for this reason, were not effective nucleation centers. An increase in the concentration of silver (3 wt %) did not increase the degree of crystallinity. This indicated that only very small amounts of silver nanoparticles can increase the crystallinity of fibers.

The average size of the cellulose crystallites was determined on the basis of the half-width of the peak 2θ~22.6°, characteristic of the plane (002). The average size of the pure and nanocomposite fibers crystallites was 4 nm. The presence of nanoparticles did not generally affect the size of the crystallites (Table 1). Only in the case of fibers containing unstabilized silver particles did the crystallite size decrease slightly and amounted to 3 nm. The size of silver crystallites with the fcc structure was determined on the basis of the peak 2θ~38° analysis. The size of silver crystallites in the fibers was much larger than that of the cellulose crystallites and ranged from 7 to 14 nm. The interplanar distance R in all the fibers was similar and was equal to 5.3 Å. This was due to the presence in the fibers of strong interactions between the nearby neighboring hydroxyl groups in either the same chain (intrachain) or different chains (interchain) [29].

### 3.3. FTIR of the Fibers

**Figure 4:**
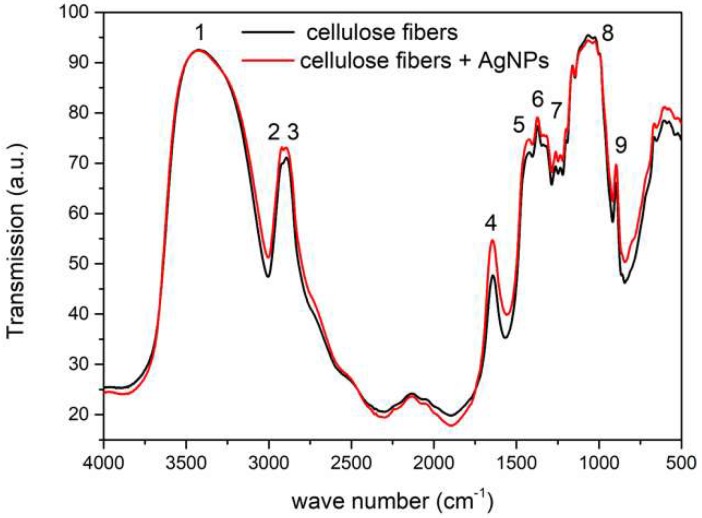
FTIR spectrum for pure cellulose fiber (black line) and the fiber doped with silver nanoparticles with PEI (red line).

The nanocomposite fibers containing 0.1% of AgNPs were characterized with Fourier Transform Infrared Spectroscopy (FTIR) to determine the effect of the nanoparticles on the structure of cellulose. Peaks characteristic of cellulose were visible in the spectra (Figure 4) [30]. The peak at 3335 cm^−1^ (1) was attributed to the OH group, and the peaks at 2900 cm^−1^ (2), 1420 cm^−1^ (5), and 1365 cm^−1^ (6) corresponded to C–H bonds. Symmetrical stretching of CH_2_ bonding was visible at 2850 cm^−1^ (3). The peak at 1635 cm^−1^ (4) corresponded to the adsorbed water. The peaks at 1200 cm^−1^ (7) and 1050 cm^−1^ (8) corresponded to the C–OH bond, and the peak at 895 cm^−1^ (9) to the C–O–C bond. In the cases of the reference fiber and the fiber doped with silver, the same peaks were visible. This confirmed the fact that silver in the fibers was in its metallic form and not in the form of potential metalorganic compounds. Additionally, in the spectrum, peaks at 1398 cm^−1^ and 1120 cm^−1^, which corresponded to metal–oxygen and metal–OH bonds, were not visible [31].

### 3.4. Release of Silver Nanoparticles

In order to determine the release of silver nanoparticles from the fibers, a washing test was carried out on a cellulose fiber containing 3% of AgNPs. The process was repeated 30 times. The solution after 10, 20, and 30 washing cycles was subjected to atomic absorption spectrometry (ASA) analysis for its silver content.

**Figure 5:**
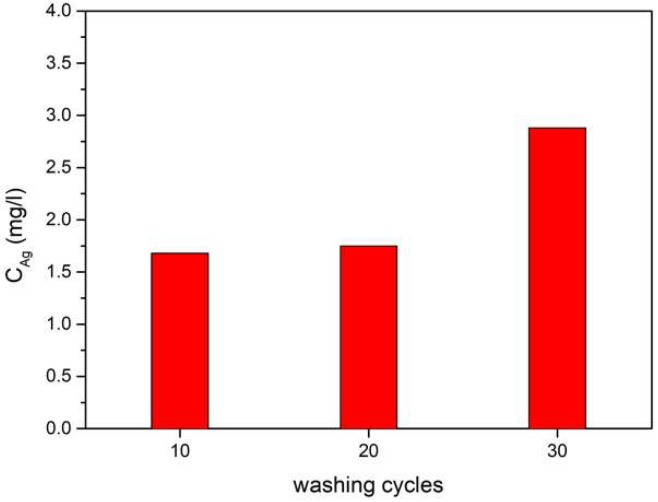
Atomic absorption spectrometry (ASA) of solutions obtained after home laundry washing of cellulose fibers containing 3 wt % AgNPs.

The obtained results showed that during the washing process, very small amounts of silver nanoparticles were released, on the limit of the method’s detection (Figure 5). Even after 30 washing cycles, less than 3 mg/L (ppm) of silver was found in the washing solution. In addition, after the washing and drying of the fibers, no color changes were observed. These results showed that in spite of the lack of chemical connections (Figure 4) between the silver nanoparticles and the cellulose, the nanoparticles were permanently placed in the volume of the fibers.

### 3.5. Flammability

The flammability of the fibers was estimated on the basis of the results from a pyrolysis combustion flow calorimeter (PCFC). This method allows the determination of flammability parameters such as heat release rate (HRR), time, and temperature of HRR (t, T) and total heat release (THR). This method is commonly used to assess flammability.

**Figure 6:**
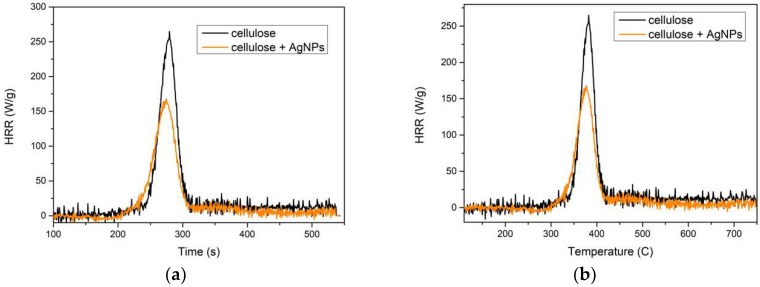
Heat release rate (HRR) of cellulose fibers: (**a**) time dependence, (**b**) temperature.

The PCFC results for the fibers (Figure 6) indicated that both types of fibers underwent a one-stage combustion process and that the silver nanoparticles reduced flammability. The HRR of fibers containing 3% of AgNPs decreased by 36%, the temperature dropped by 1.6%, the time to reach HRR decreased by 3%, and the total amount of heat released (THR) decreased by 32%. The flammability parameters of the fibers are presented in Table 2. The obtained values of HRR and THR for unmodified fibers were close to the values reported in the literature for cellulose fibers [32].

### 3.6. TG of Cellulose Fibers

**Figure 7:**
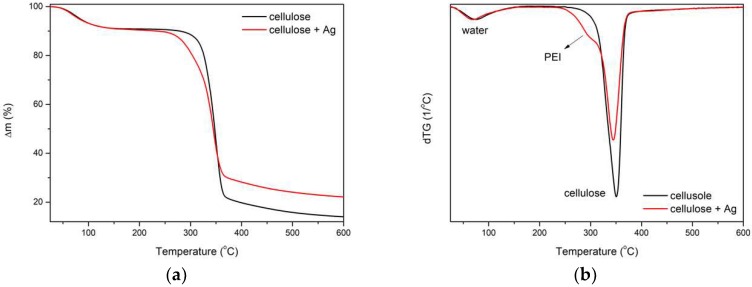
Results for cellulose fibers: (**a**) TG curve, (**b**) the DTG curve.

The evaluation of the thermal properties of the fibers was carried out using the thermogravimetric method. The comparison of the thermogravimetric (TG) and first derivative of TG (i.e., DTG) curves for non-doped fibers containing 3% of AgNPs showed that both materials had a comparable amount of adsorbed moisture. Fibers containing AgNPs had a lower temperature of degradation than those without an inorganic additive. This is related to the decomposition of the stabilizing polymer (PEI), whose maximum decomposition temperature is about 311 °C [33]. Cellulose degradation is most rapid at a temperature of about 344 °C for both materials, but this process is less intense in the case of nanocomposite fibers. Differences in the total loss of mass indicated the amounts of additives (silver and stabilizer) in the doped material, which reached about 5% by weight (Figure 7).

**Figure 8:**
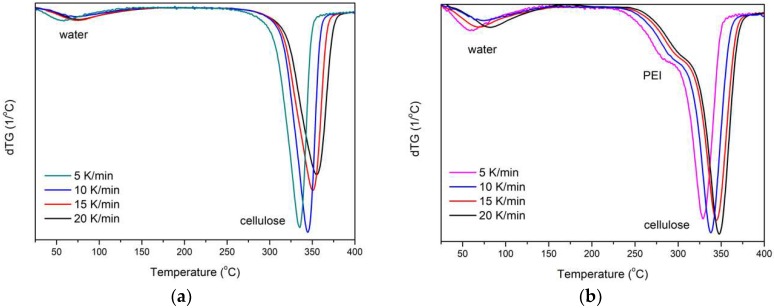
DTG curves at different heating speeds of fibers: (**a**) undoped; (**b**) doped.

The activation energy of the fiber decomposition process was determined using isoconversion linear methods, i.e., Kissinger–Akahira–Sunose (KAS) and Flynn–Wall–Ozawa (FWO) methods [34,35]. For this purpose, thermogravimetric studies of the samples were carried out at different heating rates (5, 10, 15, 20 °C/min). The results obtained in the form of the first derivative of mass loss at temperature (DTG) clearly showed that as the heating speed was increased, the maximum peak shift towards higher temperatures, as well as a reduction in peak width and increase of its intensity, were observed (Figure 8).

**Figure 9:**
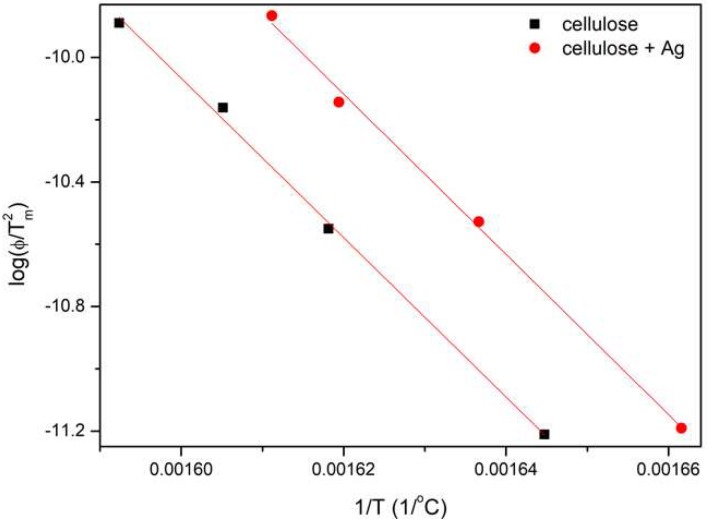
Plot ln(ϕ/*T*_m_^2^) = *f*(1/*T*_m_) for the Kissinger–Akahira–Sunose (KAS) method.

The activation energy was determined according to the KAS method. In this method, on the basis of exothermic peaks, the relationship of ln(ϕ/*T*_m2_) = *f*(1/*T*_m_) was plotted (Figure 9). According to the Coats–Redfern equation [36], the activation energy value is proportional to the slope of the line (Equation (5)):

(5) $$E_{a}=a\cdot R\;\left[\frac{\text{kJ}}{\text{mol}}\right]$$

**Figure 10:**
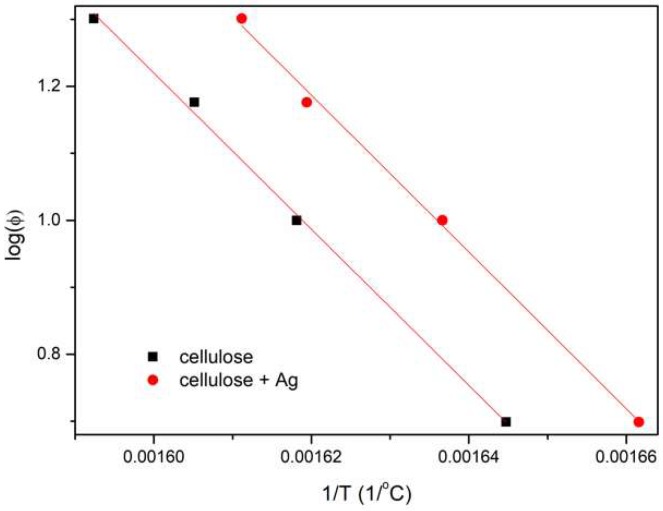
Plot of log(ϕ) = *f*(1/*T*_m_) for the Flynn–Wall–Ozawa (FWO) method.

In the FWO method, Doyle approximation [37] is taken into account, and the relationship log(ϕ) = *f*(1/*T*_m_) is plotted (Figure 10). According to this method, the activation energy is proportional to the slope of the regression line (Equation (6)):

(6) $$E_{a}=\frac{a\cdot R}{0.4567}\;\left[\frac{\text{kJ}}{\text{mol}}\right]$$

The comparison of the results showed that the activation energy of non-doped fibers was 212 kJ/mol, and that of doped fibers was 213 kJ/mol (Table 3). This indicates that the addition of silver nanoparticles did not cause a significant increase in *E_a_* at the point of the maximum cellulose decomposition rate.

Due to the fact that the flammability tests showed that the doped fibers were less flammable, it was additionally determined how the activation energy value changed as a function of material conversion (*α*). This allowed the changes of samples in the entire temperature range to be traced. The model-free kinetics developed by Vyazovkin [38] was used. The theory is based on the assumption that the activation energy is a function of material conversion *E*(*α*) (Equation (7)) and that this function is constant for a certain value of conversion *α* (iso-conversional method):

(7) $$\frac{d\alpha }{dt}=A\;exp\left(\frac{-E_{a}}{RT}\right)f\left(\alpha \right)$$

When analyzing the entire range of sample conversions, it was seen that up to about 5% of moisture evaporated with a similar *E_a_* value for both materials. A further temperature increase caused cellulose degradation.

In the case of nanocomposite fibers, PEI was first degraded to about *α* = 10% with an activation energy of about 140 kJ/mol. After this step, *E_a_* gradually increased with an increasing conversion to about 150 kJ/mol and subsequently increased again to around 220 kJ/mol. This was related to the beginning of cellulose decomposition, and the value of Ea was close to that calculated when using the KAS and FWO methods. This value of activation energy remained constant up to *α* = 70% and then started to gradually increase. The decomposition products formed mill scale on the surface of the fiber, which blocked the transport of heat and gaseous products to the atmosphere. The total degradation of the polymer occurs at about 83% conversion and was associated with an initial rapid increase and followed decrease of *E_a_*. Such a rapid process was most likely related to the thermal degradation of mill scale and the rest of cellulose.

**Figure 11:**
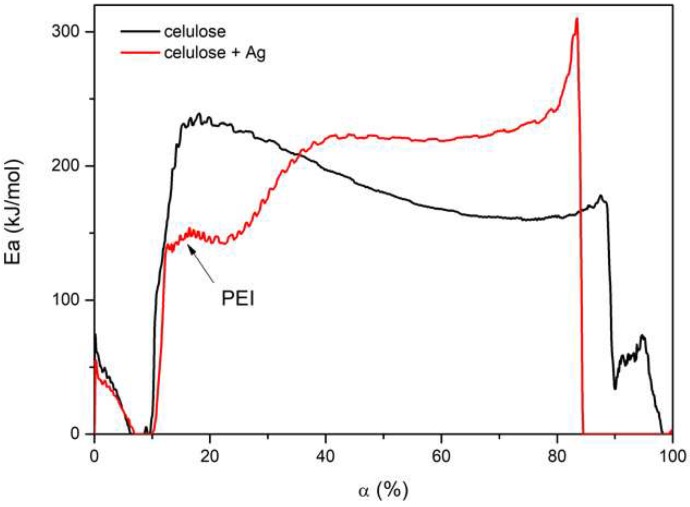
Ea changes as a function of material conversion (α).

The decomposition kinetics of undoped fiber was different. Cellulose decomposition began with an energy of approximately 230 kJ/mol. This value gradually decreased, which means that the thermal degradation of the material became easier. In the final stage, around *α* = 76%, the *E_a_* value slightly increased to 176 kJ/mol. This may also be related to the formation of mill scale on the surface of the material, although its thickness was much smaller and it was more easily degraded (Figure 11).

**Figure 12:**
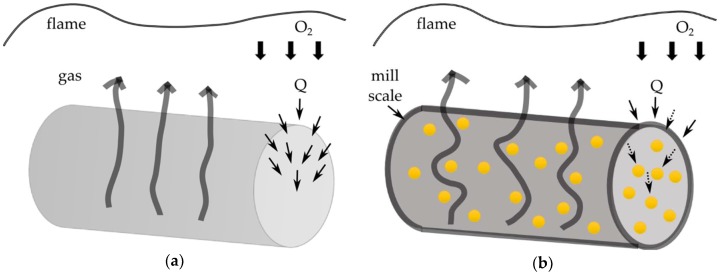
Flammability diagram of fibers: (**a**) undoped; (**b**) with AgNPs.

These results are consistent with those of the flammability tests, which showed that the addition of silver did not reduce the temperature at which HRR was observed, although its value was reduced. This confirms the thesis that the additive affected the formation of mill scale. In addition, the inorganic particles in the nanocomposite blocked the flow of heat and gases (Figure 12).

### 3.7. Electric Characterization of Fibers

Typically, the resistivity of materials is determined using Formula (8), based on the knowledge of the resistance of a sample with known dimensions. According to the SI system, the unit of resistivity is Ω·m.

(8) $$\rho =R\frac{A}{l},$$

*R* is the resistance of the sample, *A*–is cross-sectional area of the sample, *l* is the length of the sample.

**Figure 13:**
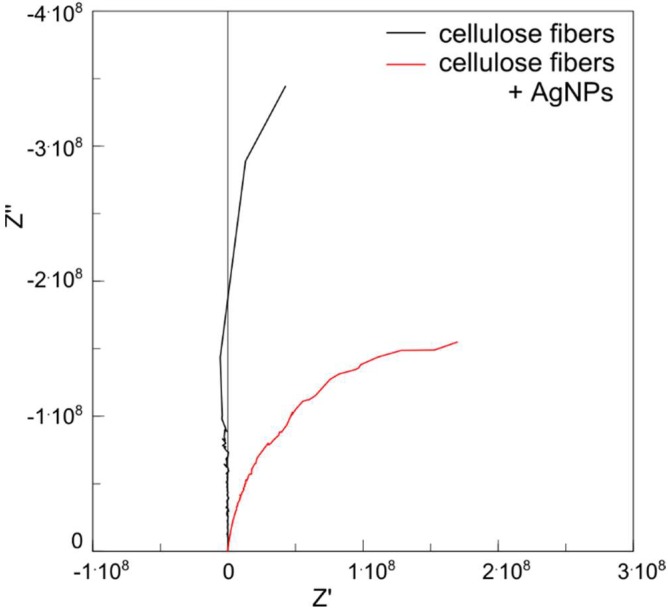
Impedance spectroscopy measurement of cellulose fibers.

Resistivity, which is widely recognized as a material constant, is easy to determine for materials with well-defined geometrical dimensions. However, in the case of specific materials such as yarns, giving the actual cross-sectional area, it is not simple to determine R. For this reason, the electrical properties of fibers are often described by providing a linear resistance value (Rl), which is the average resistance of a fiber with the length of one meter and is given in Ω/m [39]. Usually, the measurement of the value of R1 is based on the measurement of the resistance of a fiber section with a length of 1 cm, and therefore this value is most often given in Ω/cm. Investigations of the electrical properties of the fibers were made using the alternating current (AC) method (impedance spectroscopy, SI) on 1 cm-long fibers, the ends of which were placed between two flat, copper electrodes and compressed. The result of the SI measurement for unmodified and silver nanoparticles are presented in the form of the Bode plot (Figure 13).

**Figure 14:**
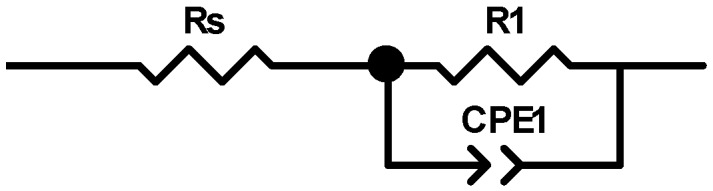
Electrical equivalent circuit. *R*s, resistance of electrical leads, *R*_1_, linear resistance of the sample, CPE, constant phase element.

On the basis of the obtained results, an electrical equivalent circuit is proposed (Figure 14) consisting of three elements: resistance of electrical leads (Rs), linear resistance of the sample (R_1_), and constant phase element (CPE) containing two components, i.e., capacitance (CPE-T) and the parameter of proximity to capacitance or resistance (CPE-P). When CPE-P is 1, this element performs as an ideal capacitor; when CPE-P is 0, it performs as an ideal resistor. The parameters of the individual elements of an equivalent circuit are given in Table 4.

The linear resistance of fibers containing 3% of silver nanoparticles was 10^8^ Ω/cm, and the linear resistance of non-doped fibers of up to eight orders of magnitude was higher, i.e., 10^16^ Ω/cm. This is a very good result considering the fact that the mechanical properties of the fibers were not weakened by doping [20].

### 3.8. Antimicrobial Properties of the Cellulose Fibers

In the present study, the four microbes *S. aureus*, *E. coli*, *A. baumannii*, and *C. albicans* were chosen as the test microorganisms because they are some of the most antibiotic-resistant human pathogens. In the last 60 years, antibiotics have been critical in the fight against infectious diseases caused by bacteria and other microorganisms. However, pathogenic microorganisms that have become resistant to antibiotic therapy are an increasing public health problem. The development of microorganism resistance has resulted in the search for new antimicrobial compounds in order to maintain a pool of effective drugs at all times. The antimicrobial effectiveness of silver nanoparticles against various microorganisms, including bacteria, fungi, and viruses, has been described by researchers [40,41]. Although AgNPs have been shown to be effective against more than 650 pathogenic microorganisms, the exact mechanism of their action is not clearly known and is a debated topic. Nevertheless, some of the basic antimicrobial effects of AgNPs have been recognized [42,43,44]. It is considered that the antimicrobial activity of silver nanoparticles includes four well-defined mechanisms: (1) adhesion of AgNsP to the cell wall and cell membrane, (2) penetration of AgNPs into the cell and damage to intracellular structures (mitochondria, vacuoles, ribosomes) and biomolecules (proteins, lipids, and DNA), (3) induction of cell toxicity and oxidative stress by AgNPs through the generation of reactive oxygen species (ROS) and free radicals, and (4) modulation of signal transduction pathways.

**Figure 15:**
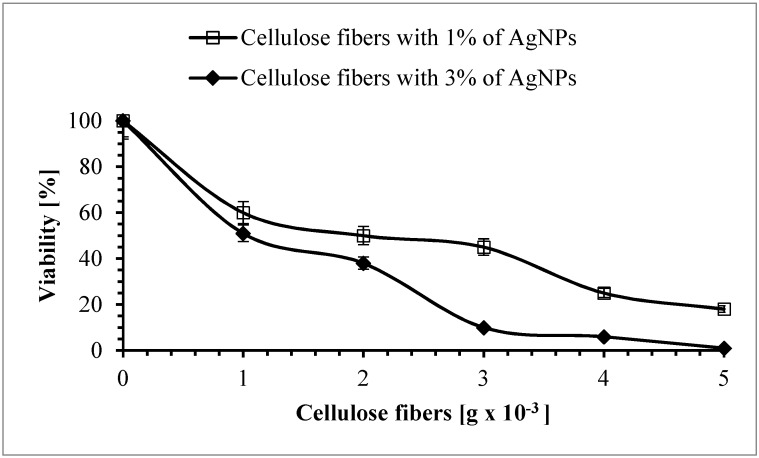
The effect of cellulose fibers modified with AgNPs on the viability of *Staphylococcus aureus*.

As shown above, silver nanoparticles were evenly distributed across the entire volume of the studied cellulose fibers and were also found on their surface, suggesting excellent antimicrobial properties. The conditions defined in our studies demonstrated that the fibers modified by AgNPs reduce in vitro the concentration of *S. aureus*, *E. coli*, *A. baumannii* and *C. albicans* viable cells in planktonic cultures. On the other hand, it was confirmed that the unmodified fibers did not show antimicrobial activity against the studied microorganisms. The number of cells after 5 h of incubation with unmodified cellulose fibers was within the measurement error (below 5%) (data not shown). Therefore, it can be concluded that the biocidal efficiency of the modified cellulose fibers was related to the presence of the silver nanoparticles. Generally, the antimicrobial activity of the studied fibers depended on the concentration of AgNPs, and it was shown that the samples containing 3% AgNPs showed higher antimicrobial activity than those with 1% of AgNPs. Moreover, the biocidal efficiency of the examined fibers depended on the type of microorganism. As can be seen in Figure 15, after 5 h of incubation with the fibers containing 1% of AgNPs, the viable count of *S. aureus* showed a reduction of 40%, 50%, 55%, 75%, and 82% for 1 × 10^−3^ g, 2 × 10^−3^ g, 3 × 10^−3^ g, 4 × 10^−3^ g, and 5 × 10^−3^ g of fibers used, respectively. The highest amount of antibacterial properties was found for 5 × 10^−3^ g of fibers containing 3% of AgNPs, and the reduction in viability of *S. aureus* was 99%.

**Figure 16:**
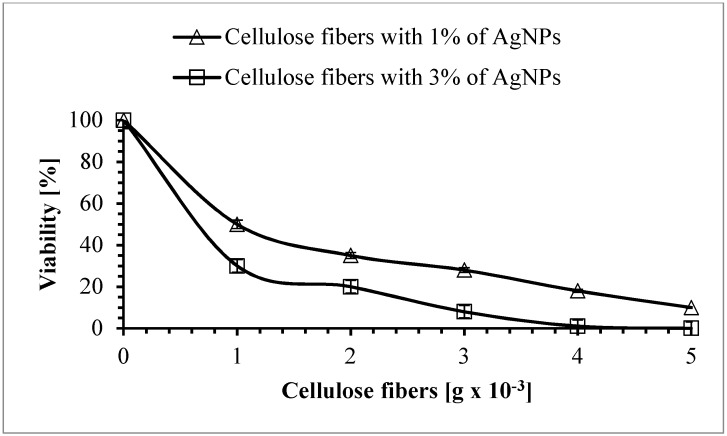
The effect of cellulose fibers modified with AgNPs on the viability of *Escherichia coli*.

A very high reduction in the number of live *E. coli* cells was observed after 5 h of exposure to the studied modified fibers (Figure 16). It was found that a reduction in viability of the cells was 70%, 80%, 92%, and 99% for 1 × 10^−3^ g, 2 × 10^−3^ g, 3 × 10^−3^ g, and 4 × 10^−3^ g of fibers containing 3% of AgNPs, respectively. When *E. coli* was treated with 5 × 10^−3^ g of these fibers, a lethal effect was observed (the number of remaining bacteria was below the detection level). It should be noticed that 5 × 10^−3^ g of fibers containing 1% of AgNPs was enough to kill 90% of these bacterial cells.

**Figure 17:**
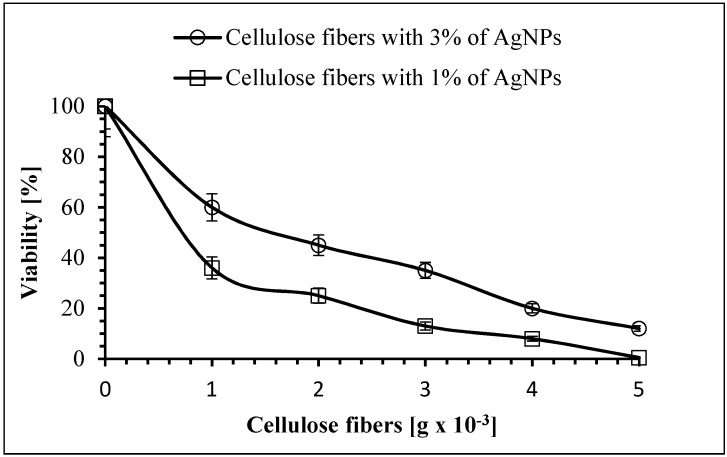
The effect of the cellulose fibers modified with AgNPs on the viability of *Acinetobacter baumannii*.

As can be seen in Figure 17, after 5 h of incubation with the fibers containing 1% of AgNPs, the viable count of *A. baumannii* showed a reduction of 40%, 55%, 65%, 80%, and 88% for 1 × 10^−3^ g, 2 × 10^−3^ g, 3 × 10^−3^ g, 4 × 10^−3^ g, and 5 × 10^−3^ g of fibers, respectively. The higher effectiveness of biocidal activity was observed when the cellulose fibers containing 3% of AgNPs were used in the experiments, and the cell death rate was 64%, 75%, 83%, and 92% for 1 × 10^−3^ g, 2 × 10^−3^ g, 3 × 10^−3^ g, and 4 × 10^−3^ g of fibers, respectively. When *A. baumannii* was treated with 5 × 10^−3^ g of these fibers, the reduction in the number of bacterial cells was 99.5%.

**Figure 18:**
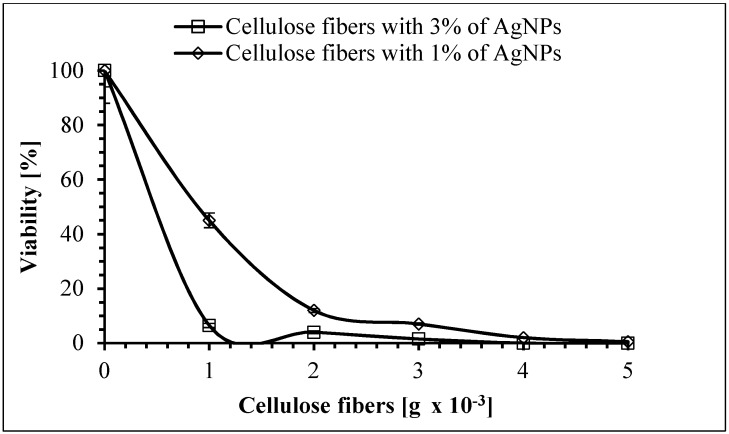
The effect of cellulose fibers modified with AgNPs on the viability of *Candida albicans*.

The next microorganism treated with the silver-modified fibers was *C. albicans*. The antifungal activity of AgNPs has previously been studied against different *Candida* species: *C. albicans*, *Candida glabrata*, and *Candida tropicalis*, and it was found that these nanoparticles completely inhibited the growth of yeasts [45]. Furthermore, it was reported that AgNPs damage the structure of the cell membrane in *C. albicans*, producing “holes” on the surface of the cells and thus inhibiting the budding process [46]. Moreover, the production of ROS, DNA fragmentation, and apoptosis has been reported [47]. Our research confirmed the excellent antifungal activity of the modified cellulose fibers. As can be seen in Figure 18, the fungicidal effectiveness of these fibers depended on the concentration of silver nanoparticles.

A very high reduction in the number of live *C. albicans* cells was observed after 5 h of exposure to the studied fibers (Figure 18). When 1 × 10^−3^ g, 2 × 10^−3^ g, 3 × 10^−3^ g, 4 × 10^−3^ g, and 5 × 10^−3^ g of fibers modified with 1% of AgNPs were used in the experiments, it was found that the reduction in cell viability was 55%, 88%, 93%, 95%, and 99.5%, respectively. An exceptionally strong fungicidal efficiency was observed when the studied yeast was treated with the samples containing 3% of AgNPs. As can be seen in Figure 18, after 5 h of incubation with these fibers, the viable count of *C. albicans* showed a reduction of 93.5%, 96%, and 98.5% for 1 × 10^−3^ g, 2 × 10^−3^ g, and 3 × 10^−3^ g of fibers, respectively. A lethal effect was observed (the number of living cells was below the detection level) when 4 × 10^−3^ g or 5 × 10^−3^ g of the modified fibers was added to the cell suspensions.

**Figure 19:**
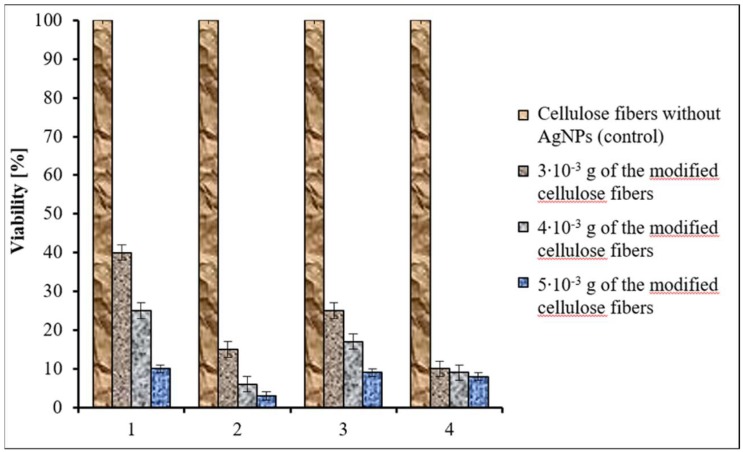
The effect of the cellulose fibers modified with 3% of AgNPs after 30 laundering cycles on the viability of *S. aureus* (1), *E. coli* (2)*, A. baumannii*, (3) and *C. albicans* (4).

The antimicrobial properties after 30 consecutive laundering cycles were tested to evaluate the laundering durability of the modified cellulose fibers, and the obtained results are shown in Figure 19.

As can be seen in this figure, after 30 consecutive laundering cycles the viable count of *S. aureus* showed a reduction of 60%, 75%, and 90% for 3 × 10^−3^ g, 4 × 10^−3^ g, and 5 × 10^−3 ^ g of fibers containing 3% of AgNPs, respectively. Under the same experimental conditions, the cell mortality of *E. coli* was 85%, 94%, and 97%. The reduction in the number of *A. baumannii* cells was 75%, 83%, and 91% when bacterial suspensions were treated with 3 × 10^−3^ g, 4 × 10^−3 ^ g, and 5  10^−3^ g of these fibers, respectively. A very high reduction in the number of live *C. albicans* cells was found even after 30 consecutive laundering cycles. It was observed that the reduction in viability of the cells was 90%, 91%, and 92% when 3 × 10^−3^ g, 4 × 10^−3^ g, or 5 × 10^−3^ g of fibers modified with AgNPs were used in the experiments. Taking into account the results of our research, it was concluded that the biocidal efficiency of the studied fibers after 30 consecutive laundering cycles decreased slightly; however, the samples still showed satisfactory antimicrobial properties.

Moreover, the obtained results showed that Gram-negative bacteria (*E. coli* and *A. baumannii*) were more sensitive to the studied modified cellulose fibers than Gram-positive bacteria (*S. aureus*). A particular sensitivity of Gram-negative bacteria to silver nanoparticles was described earlier [44,48,49]. Researchers explain this phenomenon in different ways. Some authors believe that the difference in the organization of the cell wall results in greater sensitivity of Gram-negative bacteria. It is well known that the bactericidal activity of AgNPs is affected by the thickness and composition of the cell wall of microorganisms. In Gram-positive bacteria, the cell wall consists of a negatively charged peptidoglycan, and this layer is thicker in Gram-positive bacteria than in Gram-negative bacteria. The thicker cell wall of Gram-positive bacteria, as well as the negative charge of peptidoglycan, causes silver ions to remain stuck to the cell surface. For this reason, the bactericidal effect of silver ions in *S. aureus* is prevented, and the coccus is more resistant to AgNPs [45]. Gram-negative bacteria are more susceptible to AgNPs because of a smaller cell wall thickness and lower peptidoglycan content [49]. In addition, these bacteria contain lipopolysaccharides (LPS) in the outer cell membrane, which contributes to the structural integrity of the membrane. However, LPS negative charge promotes adhesion of AgNPs and makes the bacteria more sensitive to silver nanoparticles. Several studies showed adhesion and deposition of AgNPs on the surface of bacterial cells, in particular Gram-negative bacteria [48]. Raffi et al. [49] demonstrated that the cell membrane of *E. coli* cells was completely disrupted after a few minutes of contact with AgNPs. The cell wall became peripheral, and numerous electron cavities were found at sites of damage induced by AgNPs [42]. In addition, the interaction of AgNPs with proteins containing sulfur caused irreversible changes in the cell wall structure [50]. These changes resulted in an increase in membrane permeability, which affected the ability of the cells to regulate transport activity through the plasma membrane [51]. It was proved that AgNPs impair the uptake and release of phosphate ions in *E. coli* [51]. Similarly, silver ions can also alter the transport and release of potassium (K^+^) ions from microbial cells. The increase in membrane permeability may have more pronounced effects, such as loss of cellular content through leaking, including proteins, sugars, and ATP [46,52]. Proteomic data on microbial cells treated with AgNPs showed the accumulation of immature membrane precursor proteins that destabilize the outer membrane of *E. coli* [53]. The translocation of precursor proteins into the cell membrane requires energy from the proton motive forces and ATP, and therefore the accumulation of immature precursor proteins suggests dissipation of the proton motive forces and the exhaustion of cellular ATP. This exhaustion of cellular ATP may be due to leakage or the inhibition of ATP synthesis [49,53,54]. When AgNPs penetrate microbial cells, they can interact with cell structures and molecules. For example, the interaction of AgNPs with ribosomes leads to their destruction, which in turn results in the inhibition of protein synthesis [55,56]. It has also been shown that Ag^+1^ ions bind to the thiol groups of proteins, which results in protein inactivation [56]. Previously, it was also observed that AgNPs alter the 3D structure of proteins upon interaction and interfere with disulfide bonds, blocking active binding sites, which leads to overall functional defects in the microorganisms [57]. Bhattacharya and Mukherjee [57] demonstrated that inhibition of sugar metabolism by AuNPs is due to the inactivation of the enzyme phosphomannose isomerase. This enzyme mediates the isomerization of mannose-6-phosphate into fructose-6-phosphate, the latter being an important intermediate in the glycolytic cycle. Microbial cells exposed to AgNPs also suffer from genetic alterations, such as condensation of genetic material [37]. It is well known that the DNA has sulfur and phosphorus as its major components and that AgNPs can act on these soft bases and destroy the DNA. The interaction of AgNPs with sulfur and phosphorus in DNA can lead to problems in DNA replication. As a consequence, several important cellular functions can be inhibited, which ultimately leads to cell necrosis and death [58,59].

It has also been found that nanoparticles can modulate signal transduction in bacteria. It is a well-established fact that the phosphorylation of protein substrates in bacteria influences bacterial signal transduction. Dephosphorylation is noted only in tyrosine residues of Gram-negative bacteria. The phosphotyrosine profile of bacterial peptides is altered by the nanoparticles, which leads to signal transduction inhibition and thus to the inhibition of bacterial growth [60].

It should be noted that *C. albicans* showed a high sensitivity to the studied modified fibers. It is well known that the cell walls of fungal cells have a structure that is intermediate in permeability between those of Gram-positive and Gram-negative bacteria. The outer part is a moderately porous layer of ß-glucan and mannan polysaccharides. 

The results obtained by Vazquez-Muñoz et al. [61] revealed a high accumulation of AgNPs outside the cells. However, smaller nanoparticles were also localized throughout the cytoplasm. Energy-dispersive spectroscopy analysis confirmed the presence of intracellular silver. From the obtained results it is assumed that AgNPs do not penetrate the cell, but instead release silver ions that infiltrate into the cell, which in turn leads to the formation of nanoparticles through the reduction of organic compounds present in the cell wall and cytoplasm. This phenomenon can be caused by the high fungicidal effectiveness of the studied cellulose fibers modified with AgNPs.

## 4. Conclusions

In summary, multifunctional nanocomposite cellulose fibers were obtained as a result of the direct synthesis of silver nanoparticles in a spinning solution. As a result of the modification of silver nanoparticles, the fibers acquired antibacterial properties, their flammability was reduced, and their electrical parameters were changed. Thanks to the direct synthesis of nanoparticles in the spinning solution, the nanoparticles were evenly spaced and did not form agglomerates, which had a direct impact on the mechanical strength of the fibers [20].

The presence of silver nanoparticles in fibers changed their color from yellow to reddish brown. The color change of the fibers is related to the optical properties of noble metal nanoparticles. In the case of silver nanoparticles, the peak of the localized surface plasmon resonance occurs at a wavelength around 420 nm and is responsible for their yellow color.

XRD analysis showed that a small amount of silver nanoparticles can increase the crystallinity of fibers. AgNPs in fibers are in the fcc structure. FTIR results confirmed the fact that silver in the fibers was in metallic form and not in the form of metaloorganic compounds. The results of tests of solutions after 30 cycles of hand washing confirmed that, despite the lack of chemical connections between the silver nanoparticles and cellulose, the nanoparticles were permanently placed in the volume of the fibers and practically were not rinsed out.

The modification of fibers with silver nanoparticles, which have high electrical conductivity, allowed the fiber resistance to be reduced by up to eight orders of magnitude, reaching the value of 10^8^ Ω/cm. Fibers with this level of resistance are used as antistatic fibers. In addition, these fibers are used as a core in fibers coated with high-conductive silver layers [62].

The presence of silver nanoparticles in the fibers significantly reduced their flammability by more than 30%. This is related to the presence of an inorganic component in the polymer fiber, which impedes the emission of gas combustion products and the formation of scale on the surface of the fibers and has blocking properties in relation to heat and gaseous combustion products.

The studied modified cellulose fibers showed a significant biocidal activity. It was observed that Gram-negative *E. coli* and *A. baumanni* bacteria were very sensitive to AgNPs, and 5 h incubation of cells with 5 × 10^−3^ g of these fibers containing 3% of AgNPs resulted in a lethal effect (the number of living cells was below the detection level) for *E. coli* or in the reduction in the number of *A. baumanni* cells by 99.5%. In the same experimental conditions, the viability of *S. aureus* (Gram-positive bacterium) was reduced by 99%. A lethal effect was observed when 4 × 10^−3^ g or 5 × 10^−3^ g of the modified fibers were added to the planktonic culture of *C. albicans*. The biocidal efficiency of the studied fibers after 30 consecutive laundering cycles decreased slightly, but the samples still showed satisfactory antimicrobial properties.

## Figures and Tables

**Table 1:** Degree of crystallinity of cellulose fibers.

Ag Concentration [wt %]	Crystallinity Degree	L_(002)_ [nm]	Ag L_(111)_ [nm]	R [Å]
–	0.54	4	-	5.36
0.1	0.71	4	7	5.37
0.1 (without PEI)	0.58	3	14	5.33
3	0.55	4	11	5.28

Where: L_(khl)_—the average size of crystallites; R—interplanar distance.

**Table 2:** Flammability parameters of cellulose fibers. THR, total heat release.

Fibers	T max [s]	T max [°C]	HRR [W/g]	THR [kJ/g]
Cellulose	279.5	382	274.5	10.56
Cellulose + 3% AgNPs	274.5	376	167.88	7.21

**Table 3:** Activation energy values.

Fibers	*E_a_* KAS [kJ/mol]	*E_a_* FWO [kJ/mol]	*E_a śr._* [kJ/mol]
Cellulose	212.38	211.71	212.04
Cellulose + 3% AgNPs	213.56	212.72	213.14

**Table 4:** The components value of electrical equivalent circuit of nanocomposite fibers. CPE-P, proximity to capacitance or resistance, CPE-T, capacitance.

Fibers	Rs [Ω]	R_1_ [Ω/cm]	CPE1-T [F]	CPE1-P
Cellulose	920	2.18 × 10^16^	1.58 × 10^−11^	1
Cellulose + 3% AgNPs	920	2.98 × 10^8^	4.64 × 10^−11^	0.95
